# The vmPFC-IPL functional connectivity as the neural basis of future self-continuity impacted procrastination: the mediating role of anticipated positive outcomes

**DOI:** 10.1186/s12993-024-00236-z

**Published:** 2024-05-09

**Authors:** Xiaotian Zhao, Rong Zhang, Tingyong Feng

**Affiliations:** 1 Faculty of Psychology, Southwest University, No. 2, Tian Sheng RD., Beibei, Chongqing, 400715 China; 2 Key Laboratory of Cognition and Personality, Ministry of Education, Chongqing, China

**Keywords:** Procrastination, Future self-continuity, Episodic future thinking, Voxel-based morphometry, Resting-state functional connectivity

## Abstract

Procrastination is universally acknowledged as a problematic behavior with wide-ranging consequences impacting various facets of individuals’ lives, including academic achievement, social accomplishments, and mental health. Although previous research has indicated that future self-continuity is robustly negatively correlated with procrastination, it remains unknown about the neural mechanisms underlying the impact of future self-continuity on procrastination. To address this issue, we employed a free construction approach to collect individuals’ episodic future thinking (EFT) thoughts regarding specific procrastination tasks. Next, we conducted voxel-based morphometry (VBM) and resting-state functional connectivity (RSFC) analysis to explore the neural substrates underlying future self-continuity. Behavior results revealed that future self-continuity was significantly negatively correlated with procrastination, and positively correlated with anticipated positive outcome. The VBM analysis showed a positive association between future self-continuity and gray matter volumes in the right ventromedial prefrontal cortex (vmPFC). Furthermore, the RSFC results indicated that the functional connectivity between the right vmPFC and the left inferior parietal lobule (IPL) was positively correlated with future self-continuity. More importantly, the mediation analysis demonstrated that anticipated positive outcome can completely mediate the relationship between the vmPFC-IPL functional connectivity and procrastination. These findings suggested that vmPFC-IPL functional connectivity might prompt anticipated positive outcome about the task and thereby reduce procrastination, which provides a new perspective to understand the relationship between future self-continuity and procrastination.

## Introduction

Procrastination, the deliberate postponement of intended actions despite anticipated deleterious outcomes [76], manifests as a pervasive and disruptive phenomenon. It hinders various aspects of life, encompassing academic performances [3], career achievement [54], as well as physical and mental health (e.g., satisfaction) [32, 51, 72]. Therefore, it is crucial to explore potential factors that may influence procrastination. It has been found that procrastination was related to various personality traits, including neuroticism, impulsivity, and self-control [66, 76]. Notably, future self-continuity, a relatively stable personality trait, exhibits a robust negative correlation with procrastination [17, 73]. Nonetheless, the neural underpinnings of this association remain unclear.

Future self-continuity refers to the perception of psychological connectedness between one’s present self and future self [29]. When individuals perceive the sense of consistency with their future selves, they engage in the cognitive processes of mentally projecting themselves into the future and pre-experiencing anticipated events, commonly known as episodic future thinking [8]. This process contributes to their decision-making and judgmental processes, shaping their attitudes and behaviors toward future outcomes [20, 50]. Individuals with high levels of future self-continuity perceive a greater overlap with their future selves and are more inclined to prioritize long-term benefits over immediate gains [9, 10, 39, 61]. Furthermore, high future self-continuity correlates with a greater propensity for envisioned positive future scenarios [34, 56, 81]. On the flip side, the core issue underlying procrastination is whether to do it now or later [83]. Procrastination involves making choices that affect our future, highlighting a failure in self-regulation. It reveals a disjunction between one’s present and future self [73]. Moreover, the temporal decision model of procrastination (TDM) highlights the conflict between one’s present self and future self [83]. Specifically, the present self is reluctant to endure the task aversiveness and prefers to delay it, while the future self is responsible for the task’s outcome value and desires its timely execution. This trade-off between negative task-engagement and positive task-outcome is evaluated by episodic future thinking. The combination of anticipating positive outcomes and negative engagement could predict task procrastination [80]. When the perceived task aversiveness outweighs the anticipated positive outcomes, individuals are more inclined to procrastinate [82, 83]. Collectively, we assume that individuals with elevated levels of future self-continuity could anticipate more positive outcomes, thereby reducing procrastination.

Some exploratory research has been conducted on the neural basis of future self-continuity. Previous investigations have shed light on the role of cortical midline structures (CMS), including the ventromedial prefrontal cortex, anterior cingulate cortex (ACC), and posterior cingulate cortex (PCC), in relation to future self-continuity [19, 24, 25, 29, 44]. Specifically, researchers found reduced brain activity in the vmPFC when participants contemplated future events. This reduction has emerged as a predictor of the proclivity for shortsighted decisions regarding one’s future self [55]. It is acknowledged that the vmPFC is a core region implicated in episodic future thinking [1, 13, 26]. VBM studies reported that the gray matter (GM) volume of vmPFC and parahippocampal gyrus (PHC) was negatively correlated with future time perspective, a construct that encompasses individuals’ attitudes towards the future [52]. Moreover, the lesion study demonstrated that patients with vmPFC damage revealed impairments in episodic future thinking, especially the construction of future scenarios [14, 15]. In task fMRI studies, some researchers reported significant vmPFC activation when individuals anticipated positive future events [16, 71], a state associated with individuals exhibiting heightened levels of future self-continuity. Furthermore, future self-continuity pertains to the perceived connection between one’s present self and future self, potentially associated with brain regions involved in self-referential processing, notably the vmPFC [58, 69]. Individuals with elevated future self-continuity often demonstrate a preference for long-term rewards [9, 10]. According to task fMRI studies, the BOLD activity in the vmPFC exhibits a proportional relationship with the subjective value of delayed rewards [42, 63]. Therefore, the vmPFC might play a key role in future self-continuity. Similarly, another fMRI study found that participants exhibited heightened activation of the medial prefrontal cortex when evaluating stimuli linked to their present selves, and enhanced activation in the inferior parietal cortex when processing information concerning their future selves [25]. The IPL, a crucial component of the frontoparietal network (FPN) and default mode network (DMN), supports the episodic simulation of future events [12, 28, 68]. Researchers found that participants had difficulties in differentiating themselves from a highly familiar other following repetitive transcranial magnetic stimulation (rTMS) to the right IPL [77]. Hence, the IPL may engage in self-reference processing. In summary, we assume that brain regions associated with episodic prospection, self-reference, and value representation, such as the vmPFC, PHC, and IPL, appear to be linked with future self-continuity.

Additionally, researchers have discovered that episodic prospection work, which includes the vmPFC and parahippocampal gyrus, constitutes a component of the triple brain networks involved in procrastination [22, 23]. Moreover, investigations have revealed a significant correlation between the GM volumes in the vmPFC and PHC with procrastination, thus implicating the ability to envision future scenarios [41, 52]. Resting-state fMRI study also found that the regional activity of the vmPFC and the PHC was positively correlated with procrastination [84]. Taken together, we hypothesize that the brain regions of future self-continuity, such as the vmPFC, PHC and IPL, may prompt individuals to envision more future rewards, subsequently reducing procrastination.

Therefore, the current study aims to explore the neurocognitive substrates underlying the impact of future self-continuity on procrastination. We employed the free construction method to collect individuals’ spontaneously generated thoughts when anticipating and evaluating procrastination tasks [33, 80]. This method is an undisturbed observation approach that reveals individuals’ habitual use of construction strategies and captures cognitive mechanisms unbiasedly [31, 60]. Subsequently, these thoughts were categorized according to the 2 (emotional valence: positive vs negative) × 2 (imaginary direction: outcome vs engagement) model of episodic future thinking [80]. To explore the neural anatomy associations related to future self-continuity, we employed voxel-based morphometry, a robust method widely utilized for detecting structural differences in the brain [6]. Based on VBM results, we employed resting-state functional connectivity to investigate the connectivity patterns associated with future self-continuity [4]. In this study, both VBM and RSFC analyses were explorational in nature, aiming to provide a comprehensive understanding of the neural basis underlying future self-continuity, encompassing both structural and functional aspects [49]. Firstly, we collected individuals’ episodic future thinking thoughts using the free construction method. We then assessed participants’ future self-continuity scores and trait procrastination using the Future Self-Continuity Questionnaire (FSCQ) and General Procrastination Scale (GPS) [47, 74], respectively. Secondly, we conducted both VBM to explore the associations between gray matter volumes and future self-continuity, and RSFC to reveal the functional coupling of future self-continuity. Finally, a mediation analysis was performed to further testify whether the brain pathway related to future self-continuity influences procrastination through episodic future thinking.

## Methods

### Participants

A total of 114 healthy participants (82 females; M = 21.31 years, SD = 1.106) were recruited from Southwest University in China, who provided written informed consent at the beginning of the experiment. For our RSFC analysis, we established exclusion criteria for excessive head movement, specifically defined as greater than 2 mm in translation or greater than 2 angular rotations in axis [79]. And no participant in our study was excluded in the final analysis. Analysis from G-power suggests that our sample size is sufficient to detect a medium Pearson’s r effect size = 0.3 with the power of 90% (α = 0.05) [30]. All individuals were right-handed and had normal or corrected-to-normal vision. Moreover, none of them had a history of neurological or psychiatric ailments. The study was approved by the Institutional Review Board of Southwest University. All participants underwent an MRI scan prior to completing the behavioral experiments, which included the free construction paradigm, the Future Self-Continuity Questionnaire, and the General Procrastination Scale. After the study, all participants received payments for their participation.

### Measures

*Procrastination assessment* The level of procrastination was assessed using the General Procrastination Scale [47]. The GPS consists of 20 items (e.g., “In preparing for some deadline, I often waste time by doing other things.”) that prompt participants to rate statements related to procrastination on a 5-point Likert-type scale, ranging from 1 (extremely disagree) to 5 (extremely agree). A higher score indicates a higher tendency to procrastinate. The scale demonstrated sufficient internal consistency reliability with a Cronbach’s alpha coefficient of 0.82 [47].

*Future self-continuity assessment* Future Self-Continuity Questionnaire includes 10 self-report items designed to assess individuals’ future self-continuity [74]. This scale consists of three constructs including similarity to the future self (e.g., “How similar are you now to what you will be like 10 years from now?”), vividness of the future self (e.g., “How vividly can you imagine what you will be like in 10 years from now?”), and positive affect on the future self (e.g., Do you like what you will be like 10 years from now?”). Participants rated on a six-point Likert-type scale ranging from 1 to 6. The rating scale for the sub-dimension, namely similarity, is anchored with 1 (i.e., completely different) and 6 (i.e., exactly the same). The rest of the sub-dimensions including vividness and positive affect, on the other hand, are anchored with a scale of 1 (i.e., not at all) to 6 (i.e., perfectly). The higher scores on the scale indicate higher levels of future self-continuity. A previous study has indicated that the full scale has adequate reliability, with a Cronbach’s alpha coefficient of 0.85 [74].

*The free construction paradigm* To obtain participants’ episodic prospection thoughts, we adopted the free construction paradigm (https://osf.io/8u4ns/) that was confirmed to closely resemble everyday life representations, guaranteeing ecological validity [80]. Initially, all participants were instructed that procrastination involves voluntarily delay a course of action that could and should have been started or completed already [82]. Then, each participant was required to provide a minimum of five individual-specific procrastination tasks. In detail, they could report those tasks inspired by the Common Procrastination Tasks List for College Students [80], as well as provide tasks directly on their own. The tasks that met the following criteria were excluded in the current study due to the definition of procrastination [76]: (1) Activities related to leisure and relaxation (e.g., playing games and watching movies), as well as routine tasks (e.g., eating and drinking water). (2) Tasks that were beyond the students’ current abilities (e.g., running a marathon). (3) Tasks that did not require effort (e.g., call to mom). Subsequently, participants were asked to engage in imagining each chosen task for at least 1 min, allowing their thoughts and ideas to flow without any restrictions [33]. Meanwhile, they were instructed to record their thoughts using concise statements, at least three sentences per task. Next, participants rated the degree of procrastination for each selected task on a scale ranging from 1 to 5, with higher scores indicating a greater likelihood of procrastination (“Do you procrastinate this task?” 1 = not at all, 2 = almost no; 3 = occasionally; 4 = often; 5 = always [75, 82]. The total score of participants’ procrastination rating for the selected tasks was computed as the task procrastination assessment. Furthermore, to testify the validity of this measurement, we conducted a correlation analysis to examine the relationship between the score of GPS, which is a widely-used to measure levels of trait procrastination [47], and task procrastination assessment. The correlation results revealed a significantly positive correlation between GPS scores and task procrastination assessment (*r* = 0.377, *p* < 0.001), suggesting that task procrastination assessment can serve as a valid measure of procrastination.

### Coding for future-oriented thoughts toward personalized tasks

To acquire the characteristics of episodic prospection toward tasks, three independent student coders, who were psychology majors and were blind to the experimental purpose, coded the thoughts from the free construction tasks following the 2 × 2 model of episodic future thinking [80]. The 2 × 2 model of EFT comprises four dimensions: anticipated positive engagement, anticipated positive outcome, anticipated negative engagement, and anticipated negative outcome. These dimensions were defined as follows: anticipated positive engagement refers to task engagement accompanied by positive emotions (e.g., joy, relaxation, enjoyment). Anticipated positive outcome was defined as the mental simulation of the motivational outcomes that could arise from task completion (e.g., rewards or avoidance of punishments). Anticipated negative engagement encompassed thoughts concerning negative emotions associated with task engagement (e.g., boredom, frustration, aversion). Anticipated negative outcome referred to outcomes that evoke negative emotions and lead to task avoidance (e.g., severe punishments or failure). Episodic future thinking thoughts that did not fall into the above dimensions or did not indicate emotional arousal were categorized as anticipated neutral engagement and anticipated neutral outcome. For subsequent analysis, the coders calculated the average count for each dimension to obtain the final score.

To ascertain the consistency and accuracy of the coders’ judgments in capturing the dimensions encapsulated within the 2 × 2 model of EFT, we first conducted an inter-rater reliability analysis on the coding scores assigned by the three independent coders. The results demonstrated a significant correlation between the outcomes from the three coders, revealing a robust and reliable agreement in coding. Specifically, Kendall’s coefficient of concordance (W) was substantial for positive engagement (Kendall’s W = 0.902), positive outcome (Kendall’s W = 0.938), negative engagement (Kendall’s W = 0.931), negative outcome (Kendall’s W = 0.878). Eventually, the average count of episodic future thinking thoughts in each dimension of the 2 × 2 model of EFT, as coded by the three coders, was computed to determine the score for each respective dimension.

### MRI data acquisition

Both anatomical and resting-state fMRI data were acquired on a SIMENS MAGNETOM PRISMA 3 T scanner (Siemens Medical Department, Erlangen, Germany). The high-resolution T1-weighted anatomical images (voxel size = 0.5*0.5*1 mm) were obtained by a Magnetization Prepared Rapid Acquisition Gradient-Echo (MPRAGE) pulse sequence (192 slices, voxel size = 0.5 × 0.5 × 1 mm, TR = 2530 ms, TE = 2.98 ms, flip angle = 7°, FOV = 256 mm). Besides, the T2*-weighed echo-planar imaging (EPI) sequence was utilized to collect functional images (62 slices, TR = 2000 ms, TE = 30 ms, flip angle = 90°, FOV = 224 mm, voxel size = 2*2*2 mm). Throughout the fMRI scanning, participants were instructed to keep their eyes open, stay relaxed, and maintain a motionless state.

### MRI data analysis

#### Voxel-based morphometry analysis

*Preprocessing* The neuroanatomical images were preprocessed using Statistical Parametric Mapping software (SPM12: http://www.fil.ion.ucl.ac.uk/spm/software/spm12). The following procedures were implemented as follows [5]. Firstly, for better image registration of T1, the T1-weighted images were manually reoriented to align with the coordinates of the anterior commissure at the 3-dimensional spatial origin of the Montreal Neurological Institute (MNI). Secondly, the reoriented images were segmented into the cerebral spinal fluid (CSF), gray matter (GM), and white matter (WM) [7]. Thirdly, the DARTEL algorithm was utilized to acquire a group-specific template. This template was then used to warp the participants' scans onto it using the flow field, which stored the deformation information. Lastly, the images were modulated, spatially normalized, smoothed with a Gaussian kernel with full-width at half maximum (FWHM) of 6 mm, and resliced to 1.5 × 1.5 × 1.5 mm voxel size.

*Second-level modeling* To identify the brain regions associated with future self-continuity, we employed a multiple regression model. Future self-continuity scores served as the covariate of interest, while age, gender, and global gray matter volume of the participants were included as covariates of no interest [35, 46, 62]. The global GM volumes were obtained using the MATLAB script “get_totals” (http://www.cs.ucl.ac.uk/staff/g.ridgway/vbm/get_totals.m). Subsequently, an absolute threshold of 0.2 was applied for masking, and T contrasts were utilized to identify voxels that exhibited significant correlations with participants' future self-continuity scores. Gaussian random field (GRF) correction was applied with voxel-level threshold set at* p* < 0.05 and cluster-level threshold at *p* < 0.05 (two-tailed) to control for the false-positive rates.

#### Resting-state functional connectivity analysis

*Preprocessing* Resting-state fMRI data were preprocessed using the SPM12 software. The volumes were slice-timed to adjust temporal discrepancies and realigned to correct for head motion. Subsequently, the individual T1-weighted images were co-registered with the functional images, and co-registered images were then segmented into GM, WM, and CSF. These images were then normalized to the MNI space in 2 × 2 × 2 mm voxel size and smoothed using a 6 mm FWHM Gaussian kernel.

We employed the CONN-fMRI Functional Connectivity Toolbox (version 20.b: https://www.nitrc.org/projects/conn/) for denoising the data. To effectively remove physiological noise, we utilized the anatomical component-based noise correction method (aCompCor) and incorporated the top five principal components of WM and CSF signals as noise variables in the regression model [11, 57]. Specifically, we performed a segmentation of the structural images for each participant and then eroded the WM and CSF masks by one voxel. This erosion process resulted in smaller masks that minimized the partial volumes containing gray matter, which served as the noise regions of interest [21]. Besides, we conducted a regression analysis to exclude the influence of nuisance signals and head motion by regressing out the Friston 24-parameters [67]. However, considering that the effects of motion could not be completely eliminated by regression analysis [64], we implemented data scrubbing at the individual level to further exclude the head motion artifacts. Volumes exceeding the framewise displacement (FD) threshold (0.5 mm) were identified as excessive head motion, and their 1 back and 2 forward neighboring volumes were also excluded from the analysis [65]. Furthermore, we applied a band-pass temporal filter with a frequency of 0.008–0.09 Hz to extract low-frequency fluctuations from the resting-state fMRI data and linear detrending.

*Functional connectivity analysis* The functional connectivity analysis was performed using DPABI v7.0 (http://rfmri.org/dpabi; [79]). Based on the VBM results, the right ventromedial prefrontal cortex was defined as a seed region of interest (ROI) to calculate the whole brain's voxel-wise functional connectivity. In the first level analysis, Pearson’s correlations were computed between the average BOLD signal intensity from ROI and the time series of all voxels in the brain. The correlation coefficient maps were then transformed into *z*-maps by Fisher’s transformation. In the group-level analysis, a correlation analysis was applied to probe the relationship between individual-level *z*-FC maps and future self-continuity (GRF correction; voxel level:* p* < 0.005; cluster level:* p* < 0.05, two-tailed). Regions that survived GRF correction served as seed regions for further analyses. Eventually, we extracted Fisher's *z* score of functional connectivity values from the connectivity maps of the seed ROIs for subsequent analyses.

### Statistical analyses

First, to preliminarily probe the relationship between variables of interest, correlation analyses were employed to examine the relationship between anticipated positive engagement, anticipated positive outcome, anticipated negative engagement, and anticipated negative outcome within the 2 × 2 model, as well as the task procrastination assessment and measure of future self-continuity. In order to mitigate potential confounding effects, we used the number of procrastination tasks selected by each participant as a covariate of non-interest in subsequent analyses. Moreover, to explore the neural mechanism of future self-continuity on procrastination, we applied the PROCESS macro in the SPSS INDIRECT procedure to conduct a mediation model among functional connectivity (as the independent variable), anticipated positive outcome (as the mediator), and task procrastination assessment (as the dependent variable) (5000 bootstrap samples) [38].

## Results

### Behavioral results

**Fig. 1:**
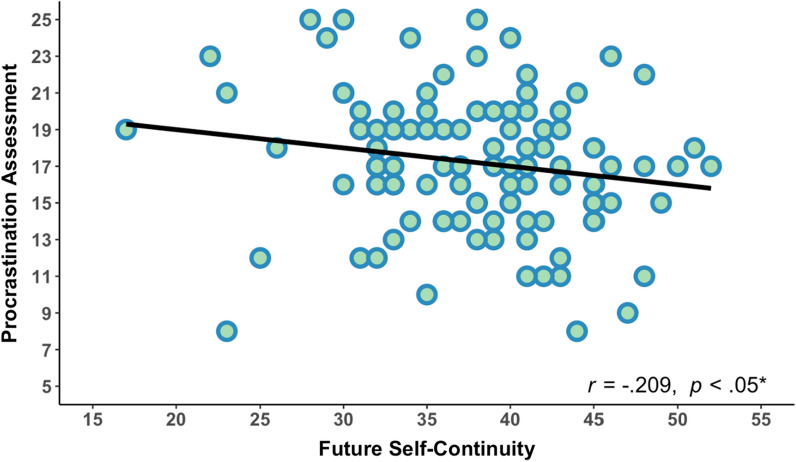
Behavioral results. Future self-continuity was negatively correlated with task procrastination assessment (*r* = − 0.209, *p* < 0.05)

The Pearson Partial correlation analysis was conducted to identify the relationship between future self-continuity, task procrastination assessment, positive engagement (PE), positive outcome (PO), negative engagement (NE), and negative outcome (NO) (see Table 1). The results revealed a significant negative correlation between future self-continuity and procrastination assessment (see Fig. 1), suggesting that higher future self-continuity was associated with lower procrastination. Notably, the intercorrelations were exclusively observed among positive outcome, future self-continuity, and procrastination.

**Table 1:** Partial Pearson correlation analysis (N = 114)

Variables	FSC	PA	PE	PO	NE	NO
FSC	1	− 0.209*	− 0.030	0.195*	− 0.039	− 0.035
PA		1	− 0.279**	− 0.273**	0.063	0.126
PE			1	0.159	0.035	− 0.306**
PO				1	− 0.187*	0.040
NE					1	− 0.117
NO						1

FSC: future self-continuity; PA: task procrastination assessment; PE: positive engagement; PO: positive outcome; NE: negative engagement; NO: negative outcome

Covariate: the number of procrastination tasks selected by each participant

Furthermore, to investigate the potential influence of age and gender on the variables, additional Pearson correlation analyses and independent sample t-tests were performed. The findings indicated that age was not significantly correlated with any variables (*r*_FSC_ = 0.051, *p* = 0.590; *r*_PA_ = 0.017, *p* = 0.856; *r*_PE_ = − 0.017, *p* = 0.854; *r*_PO_ = 0.036, *p* = 0.700; *r*_NE_ = 0.034, *p* = 0.718; *r*_NO_ = 0.039, *p* = 0.682). Additionally, no gender differences were observed in these variables: FSC,* t*_(112)_ = − 0.541, *p* = 0.590; PA,* t*_(112)_ = − 0.182, *p* = 0.856; PE,* t*_(112)_ = 0.185, *p* = 0.854; PO,* t*_(112)_ = − 0.386, *p* = 0.700; NE,* t*_(112)_ = − 0.363, *p* = 0.718; NO,* t*_(112)_ = − 0.410, *p* = 0.682.

### The VBM results

**Fig. 2:**
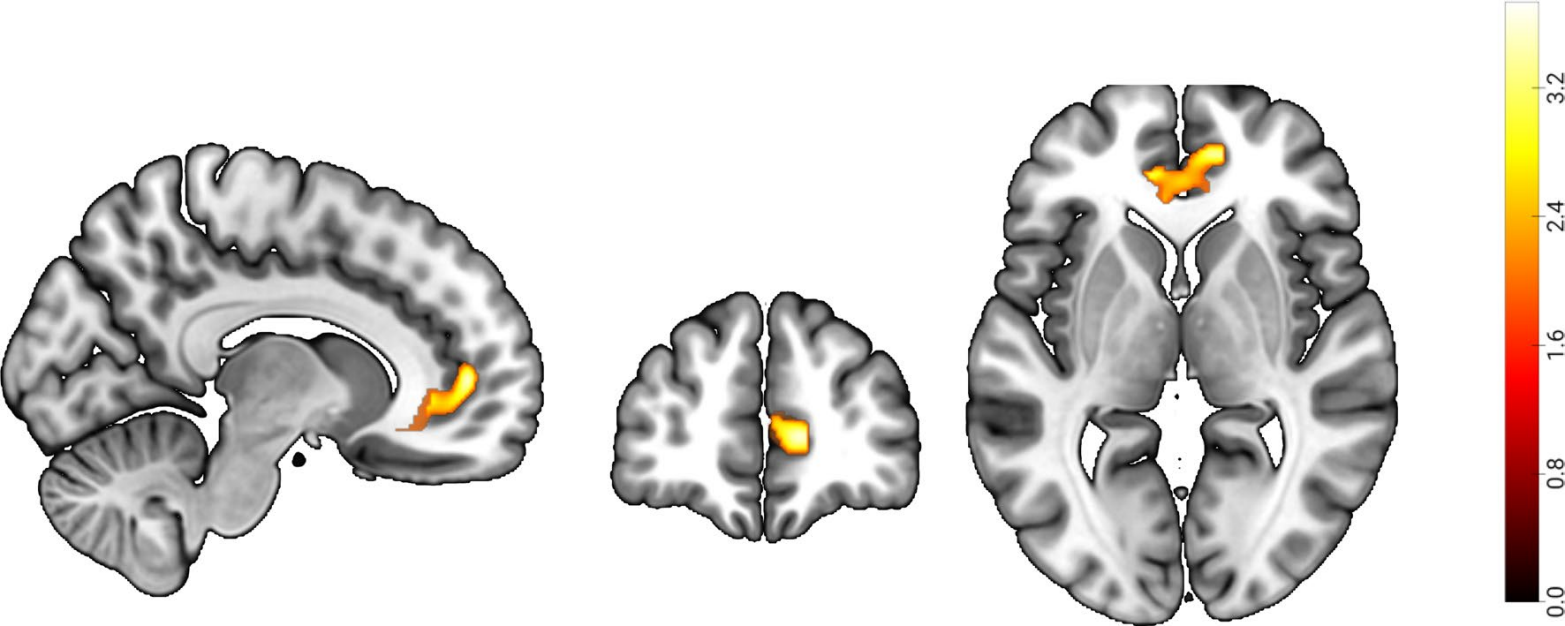
The VBM results. Future self-continuity scores were positively correlated with the GM volumes in the vmPFC (voxel significance: *p* < 0.05; cluster significance: *p* < 0.05; two tailed; GRF corrected)

To investigate the structural basis underlying future self-continuity, we adopted a multiple regression analysis to identify the association between GM volumes and future self-continuity, including age, gender, and global GM volume of participants as covariates of no interest. VBM results indicated that future self-continuity was positively associated with GM volumes in the right ventromedial prefrontal cortex (vmPFC; MNI: 12, 49.5, 4.5; voxel = 126; GRF corrected, voxel *p* < 0.05, cluster *p* < 0.05; see Fig. 2, Table 2).

**Table 2:** Brain region significantly correlated with future self-continuity

Variable	Brain region	MNI	Cluster size	t
future self-continuity	+ vmPFC.R	12 49.5 4.5	126	3.7381

### The RSFC results

**Fig. 3:**
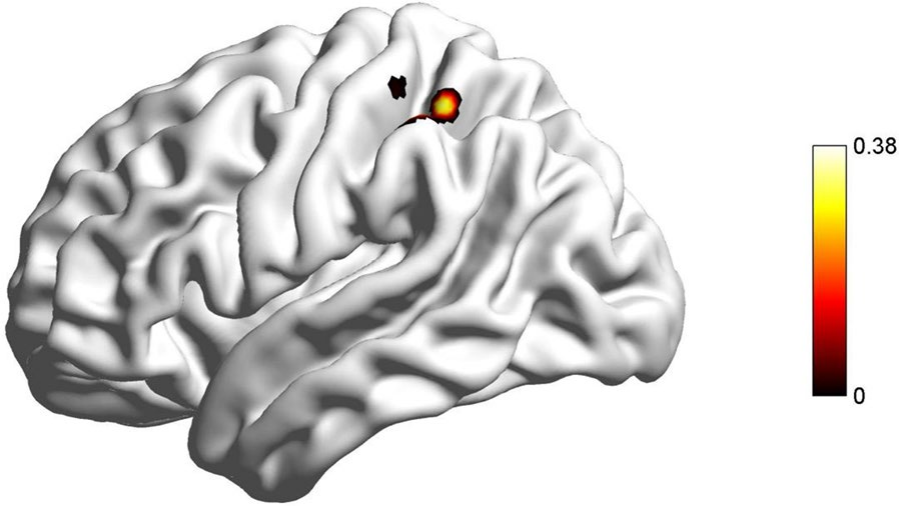
The RSFC results. Functional connectivity between the right vmPFC seed region and left IPL was positively correlated with future self-continuity (voxel significance: *p* < 0.005; cluster significance: *p* < 0.05; two tailed; GRF corrected)

Based on the VBM results, we conducted whole-brain functional connectivity analyses using the right vmPFC seed ROI derived from VBM. The results showed a significant positive association between future self-continuity and functional connectivity between right vmPFC and left inferior parietal lobule (IPL; MNI: − 50, − 36,60; voxel = 109; GRF corrected, voxel *p* < 0.005, cluster *p* < 0.05; see Fig. 3, Table 3). Furthermore, Pearson’s correlation analysis revealed a significant positive correlation between the functional connectivity of the right vmPFC and left IPL and positive outcome (*r* = 0.199, *p* < 0.05), while a significant negative correlation was observed between the functional connectivity of the right vmPFC and left IPL and task procrastination assessment (*r* = − 0.208, *p* < 0.05).

**Table 3:** Functional connectivity correlated with future self-continuity

Variable	Seed	Brain region	BA	MNI	Cluster size	r
future self-continuity	vmPFC.R	IPL.L	40	− 50 − 36 60	109	0.37846

### Mediation results

**Fig. 4:**
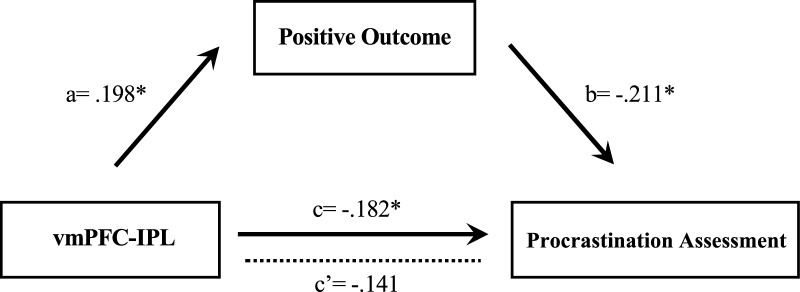
The mediated model. The mediation analysis indicated that anticipated positive outcome could completely mediate the relationship between vmPFC-IPL and procrastination assessment

To examine the potential influence of the functional connectivity responsible for future self-continuity on procrastination, mediated by anticipated positive outcome, we applied the mediation analysis by the PROCESS of SPSS [38]. After conducting 5000 bootstrap samples, the results revealed that anticipated positive outcome fully mediated the right vmPFC-left IPL functional connectivity and procrastination assessment (indirect effect estimate = − 0.1566, 95% CI [− 0.36, − 0.0051]; Fig. 4). These findings suggested that the right vmPFC-left IPL functional connectivity (correlated with future self-continuity) may influence the procrastination through anticipated positive outcome.

## Discussion

The current study aimed to uncover the neural underpinning responsible for the effect of future self-continuity on procrastination. The behavioral results indicated that future self-continuity was negatively correlated with procrastination. Furthermore, VBM analysis found a positive correlation between future self-continuity and gray matter volumes in the right ventromedial prefrontal cortex. The RSFC results demonstrated that the vmPFC-IPL functional connectivity was positively associated with future self-continuity. Moreover, the mediation analysis showed that the vmPFC-IPL coupling, which underlies the neural correlates of future self-continuity, exerted an impact on procrastination through anticipated positive outcome. Overall, these findings suggest that the functional connectivity between the right vmPFC and the left IPL may support individuals in envisioning more positive outcomes, ultimately reducing procrastination. This provides novel insights into the neural mechanism underlying the relationship between future self-continuity and procrastination.

Consistent with our hypothesis, higher future self-continuity was associated with reduced task procrastination, which can be attributed to heightened anticipations of positive outcomes. High future self-continuity individuals recognize that their present actions and decisions will have consequences for their future selves. This recognition leads to a greater sense of personal responsibility and a more proactive decision-making approach that prioritizes future rewards over immediate needs [2, 40, 45]. Based on the future self-continuity model [39], individuals with high future self-continuity tend to perceive significant similarities between their future and present selves [10]. They also exhibit more vivid mental imagery when imaging their future selves [18, 78], and hold more positive expectations pertaining to future outcomes [81]. In the context of procrastination, more episodic prospection of positive outcome could lead to an increase in outcome value of the task, ultimately leading to reduced procrastination behavior [83]. Our investigation aligns with earlier finding on episodic future thinking, which found that the anticipation of positive outcomes, a core component of EFT, is crucial in the reduction of procrastination [80]. We complement this by demonstrating that high future self-continuity individuals can anticipate more positive outcome, thus mitigating procrastination behaviors. Furthermore, Liu et al. [52] revealed that future time perspective negatively correlated with procrastination. This evidence jointly suggests a positive future-oriented thinking plays a crucial role in reducing procrastination. Hence, individuals characterized by elevated future self-continuity may anticipate more positive outcomes associated with procrastination tasks, thereby promoting task execution and reducing procrastination tendencies.

The VBM analysis unveiled a positive correlation between GM volumes in the right ventromedial prefrontal cortex and future self-continuity. According to previous studies, the vmPFC constitutes a central neural hub engaged in episodic future thinking, self-referential processing, and the representation of subjective value [27, 42, 68]. Some research has identified a negative correlation between the volume of GM in the left vmPFC and future time perspective [52]. Our results collectively affirm the critical role of vmPFC in future-oriented thinking. Besides, Yang et al. [80] found that the left dorsolateral prefrontal cortex (dlPFC) was positively correlated with anticipated positive outcomes. Complementary to this, applying active transcranial direct current stimulation (tDCS) over the left dlPFC could increase task outcome value (Xu et al., 2023). Taken together, these findings suggest that the prefrontal cortex, especially vmPFC and dlPFC, may all be responsible for simulating future outcomes. Neuropsychological investigations focused on brain lesions have demonstrated that damage to the vmPFC impaired the ability of episodic future thinking [14, 15]. Furthermore, activation in the vmPFC amplified during the simulation of positive future scenarios, and this activation correlated with the anticipated reward magnitude of imagined experiences [13]. Besides, when individuals were faced with different value-based decision options during intertemporal choice, the vmPFC was also activated [42, 43, 48]. Consequently, our findings propose a significant involvement of the vmPFC in future self-continuity, implying a fundamental association between future self-continuity and anticipated positive outcomes.

The RSFC analysis demonstrated a positive association between the right vmPFC-left IPL functional connectivity and future self-continuity. As part of the medial temporal lobe (MTL) subsystem, the inferior parietal lobule serves as a pivotal region for the construction of imagined scenes based on detailed episodic retrieval [12, 36, 68]. Researchers have found IPL activity increased during the construction of future events [53]. Additionally, RSFC analysis demonstrated a stronger coupling between the hippocampus and the IPL after the induction of episodic specificity. This finding empirically supports that IPL is linked to episodic future thinking processes [53]. Furthermore, task-related fMRI studies unveiled that the parietal cortex exhibited a greater representation of subjective time experience [37, 59]. In a comprehensive context, a notable trait among individuals with high levels of future self-continuity is the subjective perception of cross-temporal self-consistency [70]. Moreover, other researchers found that the inferior parietal cortex played a role in discerning the current self from temporally distant selves [25]. Collectively, our RSFC analysis suggests that the connectivity between the right vmPFC and the left IPL, potentially contributing to heightened positive future outcome episodic prospection, underpins the neural basis for future self-continuity.

In line with our hypothesis, the mediation analysis indicated that the functional connectivity of future self-continuity indirectly influenced procrastination, with anticipated positive outcome serving as a complete mediating factor. According to the future self-continuity model, people with high future self-continuity tend to hold a positive outlook regarding their future selves [39]. A preceding study has shown that a strong connectedness between one’s current self and temporal self results in a more favorable valuation of the future scenarios [81]. As mentioned above, the vmPFC-IPL functional connectivity could support more anticipated positive future outcomes. Concerning the issue of procrastination, the triple brain networks of procrastination posited that the vmPFC is engaged in episodic prospection [22]. Specifically, procrastination demonstrated a positive association with spontaneous activity in the vmPFC [84]. Moreover, the integrity of white matter connectivity between the insula and IPL was found to be negatively correlated to procrastination [23]. Besides, the temporal decision model suggests that the anticipation of positive outcomes could increase the utility of future rewards for upcoming tasks, consequently reducing procrastination [82]. In summary, these findings suggest that the vmPFC-IPL functional connectivity associated with future self-continuity might increase individuals’ anticipation of positive outcomes, leading to more subjective value of future tasks and reducing procrastination.

The present study possessed certain limitations that should be acknowledged. Firstly, although we logically found the reasonable neural substrates underlying the link between future self-continuity and procrastination, our findings cannot be simply inferred as the causal effect. To delve deeper into the chain of causation, future researchers could consider employing brain stimulation studies on targeted brain regions or employing task fMRI to characterize variations in dynamic BOLD responses. The second limitation of the current study is the homogeneity of our sample, which was predominantly composed of university students. This demographic homogeneity limits the reproductivity and generalizability of our findings to the broader population. Future research should extend these findings across a wider demographic spectrum, including variations in age, educational background, and life experiences. Thirdly, our study focused exclusively on the relationship between future self-continuity and task procrastination, without extension to trait procrastination, which failed to capture the procrastination tendencies. This gap limits the depth of our understanding regarding the impact of future self-continuity on procrastination. Future studies could employ longitudinal designs to assess procrastination tendencies to fully grasp the relationship between future self-continuity and procrastination from both situational and dispositional perspectives.

In summary, our study contributes valuable evidence supporting the mediating role of anticipated positive outcome in the association between future self-continuity and procrastination. The VBM analysis found that the GM volume of the right vmPFC was significantly positively related to future self-continuity. Furthermore, the RSFC results showed that vmPFC-IPL functional connectivity was positively correlated with future self-continuity. Notably, this functional pattern exerts an impact on procrastination through anticipated positive outcome. Collectively, these findings provide novel insights for the development of interventions aimed at reducing procrastination tendencies by enhancing episodic prospection of positive outcomes.

## Data Availability

No datasets were generated or analysed during the current study.
