# Correction to: The vmPFC-IPL functional connectivity as the neural basis of future self-continuity impacted procrastination: the mediating role of anticipated positive outcomes

**DOI:** 10.1186/s12993-024-00249-8

**Published:** 2024-09-11

**Authors:** Xiaotian Zhao, Rong Zhang, Tingyong Feng

**Affiliations:** 1 Faculty of Psychology, Southwest University, No. 2, Tian Sheng RD., Beibei, Chongqing, 400715 China; 2 Key Laboratory of Cognition and Personality, Ministry of Education, Chongqing, China

**Correction to: Behavioral and Brain Functions (2024) 20:11**

10.1186/s12993-024-00236-z

Following publication of the original article [1], the author noticed an error in Results section. The correlation results for the influence of age on various variables were incorrectly provided. This error has occurred during the transcription of result which has been corrected with this correction.

In Results section under the heading “Behavioral results”, the sentence should read, “The findings indicated that age was not significantly correlated with any variables (*r*_FSC_=.087, *p* = .360; *r*_PA_=-.117, *p* = .215; *r*_PE_=-.123, *p* = .193; *r*_PO_=.078, *p* = .409; *r*_NE_=.132, *p* = .162; *r*_NO_=.153, *p* = .105)” instead of “The findings indicated that age was not significantly correlated with any variables (*r*_FSC_=0.051, *p* = 0.590; *r*_PA_=0.017, *p* = 0.856; *r*_PE_ = − 0.017, *p* = 0.854; *r*_PO_ =0.036, *p* = 0.700; *r*_NE_ =0.034, *p* = 0.718; *r*_NO_=0.039, *p* = 0.682)”.
